# Impact of switching from intravenous to oral linezolid therapy in Japanese patients: a retrospective cohort study

**DOI:** 10.1186/s40545-016-0087-1

**Published:** 2016-10-28

**Authors:** Akihiro Tanaka, Akiko Yano, Shinichi Watanabe, Mamoru Tanaka, Hiroaki Araki

**Affiliations:** Division of Pharmacy, Ehime University Hospital, 454 Shitsukawa, Toon, Ehime 791-0295 Japan

**Keywords:** Switching therapy, Linezolid, Cost

## Abstract

**Background:**

High oral bioavailability of antimicrobial agents can result in the replacement of intravenous (IV) therapy with oral therapy when a patient meets defined clinical criteria. However, few studies have evaluated the effects of switching antibiotic administration route in Japan, especially for linezolid. This study evaluated an IV-to-oral antibiotic switching program for linezolid treatment at a university hospital in Japan.

**Methods:**

In a retrospective cohort study of 73 patients, we assessed the efficacy and safety of IV-to-oral linezolid therapy (*n* = 21 patients) compared with IV therapy alone (*n* = 52 patients).

**Results:**

Duration of linezolid treatment, changes in C-reactive protein or platelet count from baseline, re-administration of anti-methicillin-resistant *Staphylococcus aureus* agent within 90 days of discharge, and mortality within 28 days of discharge were not significantly different between the two groups.

**Conclusions:**

An IV-to-oral switching program could reduce the duration of IV linezolid therapy without worsening clinical outcomes in Japanese patients receiving linezolid therapy.

## Background

Appropriate use of antimicrobial agents is important for clinical outcomes, patient safety, and minimizing drug resistance [1–3]. Stewardship programs promote judicious use of antimicrobial agents by selecting the appropriate drug, dose, duration, and route of administration [4].

The high oral bioavailability of antimicrobial agents such as fluoroquinolones, linezolid, fluconazole, and voriconazole justifies the conversion of intravenous (IV) therapy to oral therapy if a patient meets defined clinical criteria. IV-to-oral switching programs for antibiotics were implemented in several countries in the 1990s. This strategy can result in lower costs as well as reduced burden for nursing staff, prevalence of catheter-related infections, and duration of hospital stay [5, 6]. Few studies to date have evaluated the effects of switching administration routes for antibiotic therapy in Japan from IV to oral, especially for linezolid, which is why the present study was carried out. This study evaluated an IV-to-oral antibiotic switching program for linezolid treatment at a university hospital in Japan.

## Methods

### Design and study population

We conducted a retrospective cohort study of patients hospitalized at Ehime University Hospital (Ehime, Japan). The study was carried out in accordance with the guidelines for human studies adopted by the Ethics Committee of Ehime University Hospital (approval number: 1408002). Data were collected from records dating from 1 May 2009 to 30 April 2014.

We excluded patients who had received antibiotic therapy for <5 days, had neutropenic disease, or were aged <15 years. We divided patients into two groups, IV to oral therapy (IOT) and IV therapy alone (IVT). Criteria to define inappropriate IV administration of antibiotics were used in switching patients from IV to oral route. The criteria included: body temperature of 38 °C during the previous 24 h; decreased or normal blood leucocyte count; absence of unexplained tachycardia; functional gastrointestinal tract (patient could eat or had a functional gastric feeding tube); and absence of vomiting, diarrhea, or severe sepsis [7]. In each case, pharmacists from the infection control team had recommended the switching from an IV route to an oral route. Assignment of patients to either group was determined by the attending physician, who also made the decision on whether to discontinue linezolid therapy.

### Outcome measures

Several indicators were used to assess the efficacy and safety of switching from IV to oral linezolid therapy in comparison with IV therapy alone: decrease in C-reactive protein (CRP) level of ≥30 % from baseline; decrease in platelet count of ≥30 % from baseline; re-administration of an anti-methicillin-resistant *Staphylococcus aureus* (MRSA) agent <90 days after hospital discharge; and death <28 days after hospital discharge [8–10].

### Statistical analysis

Statistical analysis was performed using add-in software for Mac Statistical Analysis v2.0 (Esumi, Tokyo, Japan). To compare differences in the distribution of baseline characteristics, we used the *x* ^2^ test for binary data and Student’s *t*-test. The chi-squared test was used for discrete variables. Values of *p* < 0.05 were considered significant.

## Results

Seventy-three patients were evaluated retrospectively. Baseline characteristics of the IOT group (21 patients) and IVT group (52 patients) are shown in Table 1. Age, sex, white blood cell count, CRP, and platelet count were similar between the groups.

**Table 1:** Statistical analysis of patient characteristics in the intravenous to oral therapy and intravenous therapy groups

	Intravenous to oral therapy group (*n* = 21)	Intravenous therapy group (*n* = 52)	*p-*value
Age (years)	64 ± 19	65 ± 16	0.69
Male (n [%])	19 (90.5)	40 (76.9)	0.18
White blood cell count	8.8 ± 3.8	10.6 ± 5.7	0.20
CRP	6.9 ± 4.8	8.6 ± 6.7	0.20
Platelet count	25.5 ± 9.7	23.2 ± 11.9	0.45
Indication (n [%])			
Lower respiratory infection	7 (33.3)	13 (25.0)	0.47
Skin, soft tissue, bone or joint infection	7 (33.3)	20 (38.5)	0.68
Urinary/genital tract infection	2 (9.5)	3 (5.8)	0.57
Intra-abdominal infection	1 (4.8)	2 (3.8)	0.86
Other/unknown	4 (19.0)	14 (26.9)	0.48

*CRP* C-reactive protein; data are the mean ± standard deviation

*P*-values were calculated based on Students *t*-test or Welch’s *t*-test for continuous variables and chi-squared analysis for discrete variables

Table 2 shows the clinical outcomes of the two groups. Duration of linezolid treatment, reduction in CRP level of ≥30 % from baseline, reduction in platelet count of ≥30 % from baseline, re-administration of an anti-MRSA agent <90 days after hospital discharge, and death <28 days after hospital discharge were not significantly different between the two groups.

**Table 2:** Comparison of clinical outcomes between the two groups

	Intravenous to oral therapy group (*n* = 21)	Intravenous therapy group (*n* = 52)	*p-*value
Duration of linezolid treatment (days)	12 ± 5.8	14 ± 5.6	0.22
Reduction in level of CRP from baseline of ≥30 % (n [%])	16 (76.2)	38 (73.1)	0.78
Reduction in platelet count from baseline of ≥30 % (n [%])	7 (33.3)	20 (38.5)	0.68
Re-administration of an anti-MRSA agent <90 days after hospital discharge (n [%])	2 (9.5)	7 (13.5)	0.64
Death <28 days after hospital discharge (n [%])	3 (14.3)	10 (19.2)	0.62
Drug costs (/day/person)	US$222	US$305	<0.001

MRSA, methicillin-resistant *Staphylococcus aureus*; data are the mean ± standard deviation

*P*-values were calculated based on Students *t*-test or Welch’s *t*-test for continuous variables and chi-squared analysis for discrete variables

Switching from IV to oral linezolid therapy was associated with a reduction in drug costs (\2,540,820 [US$21,173]; average \9,964 [US$83]/day/person), as IV formulations (average \36,574 [$305]/day/person) were more expensive (*p* < 0.001) than oral formulations (average \26,610 [$222]/day/person).

## Discussion

Reductions in CRP level and platelet count of ≥30 % from baseline were not significantly different between the IOT and IVT groups. Additionally, there was no significant difference in duration of linezolid treatment, re-administration of an anti-MRSA agent <90 days after hospital discharge, or death <28 days after hospital discharge between the two groups. These results suggest that switching from IV to oral linezolid therapy might be as effective and safe as maintaining patients on IV linezolid therapy alone.

A meta-analysis showed that earlier switching to antibiotic administration via the oral route was as effective as continuing IV therapy in patients with community-acquired pneumonia [11]. Similar results have been observed for intra-abdominal infections and spontaneous bacterial peritonitis [12, 13]. In Japan, earlier switching to antibiotic administration via the oral route has been shown to result in earlier clinical stability and reduced use of unnecessary antibiotics, without worsening clinical outcomes in patients hospitalized with mild and moderate community-acquired pneumonia [14]. However, reports on various MRSA infections acquired while receiving linezolid therapy are scarce. We therefore evaluated Japanese patients with various MRSA infections receiving linezolid therapy.

In the present study, the decision to implement IV therapy and to switch from IV to oral therapy was at the discretion of the attending physician. Incidence of switching from IV to oral linezolid therapy was low (21/73, 28.8 %), likely as a result of attending physicians’ attitudes towards switching administration route in Japan [14].

The direct cost saving of switching from IV to oral linezolid therapy was \9964 ($83)/day/person. Indirect costs of IV preparation and administration have been estimated to add 13 %–113 % to the costs of drugs [15]. The direct cost saving of switching from IV to oral linezolid therapy was significant. Therefore, we recommend switching from IV to oral linezolid therapy where applicable.

Our study had some limitations. First, it was not randomized in design, and data were collected retrospectively at a single institution. Second, a small sample size was included. Furthermore, a selection bias may have been present because it was the attending physician who decided whether to switch the administration route. Finally, we did not evaluate the length of hospital stay.

## Conclusions

Switching from IV to oral linezolid therapy could reduce the duration of IV linezolid therapy without worsening clinical outcomes of Japanese patients. The direct cost saving of switching from IV to oral linezolid therapy was significant. However, our study was limited by its small sample size.

## Abbreviations

CRP: C-reactive protein
IOT: IV to oral therapy
IV: Intravenous
IVT: IV therapy
MRSA: Anti-methicillin-resistant *Staphylococcus aureus*
